# Cr Alloying Enhanced Strength–Ductility Synergy in TiZrNb Alloys at Intermediate Temperature: A Comparative Study with Al and Cu

**DOI:** 10.3390/ma19101930

**Published:** 2026-05-08

**Authors:** Yelong An, Guoqiang Liu, Yu Zhang, Bingtao Tang, Yong Zhao, Aihui Zhang, Yakai Bai, Depeng Shen

**Affiliations:** 1 School of Mechanical Engineering, Shandong Key Laboratory of CNC Machine Tool Functional Components, Qilu University of Technology (Shandong Academy of Sciences), Jinan 250353, Chinatbtsh@hotmail.com (B.T.);; 2 Shandong Institute of Mechanical Design and Research, Jinan 250031, China; 3 Shandong Dazhong Mechanical Manufacturing Co., Ltd., Jinan 271100, China

**Keywords:** TiZrNbCr alloy, Cr microalloying, intermediate-temperature tensile behavior, Zr-rich precipitates

## Abstract

A systematic investigation was conducted on the effects of Cr alloying on the tensile behavior and microstructural evolution of TiZrNb medium-entropy alloys at 673 K. For comparison, the influences of Al and Cu alloying on the mechanical properties of TiZrNb were also examined. Although Al and Cu alloying enhanced the ultimate tensile strength at room temperature, their improvements in strength and ductility at 673 K were limited. In contrast, the TiZrNb_98.5_Cr_1.5_ alloy retained a single body-centered cubic (BCC) phase without forming the conventionally expected Laves phase. Cr effectively suppressed the formation of Zr-rich precipitates. At a strain rate of 1.67 × 10^−3^ s^−1^ and 673 K, TiZrNb_98.5_Cr_1.5_ exhibits an increase in the ultimate tensile strength of approximately 408 MPa compared with the base TiZrNb alloy, while the fracture elongation increases from 10% to 25% and the threshold stress rises from 669 MPa to 1196 MPa, achieving a markedly improved strength–ductility synergy. These results indicate that Cr alloying effectively stabilizes the microstructure and enhances the mechanical performance of TiZrNb alloys at 673 K by suppressing precipitate formation and reducing dislocation accumulation, outperforming Al and Cu alloying at the same temperature.

## 1. Introduction

TiZrNb medium-entropy alloys (MEAs) exhibit pronounced lattice distortion and sluggish diffusion effects, which effectively impede dislocation motion and atomic diffusion, thereby endowing them with excellent strength and thermal stability [1,2]. In addition, these alloys generally possess a low elastic modulus and good corrosion resistance [3], demonstrating significant application potential in aerospace [4], marine engineering [5], and biomedical fields [6,7].

For optimizing the performance of TiZrNb alloys, alloying regulation has been recognized as an effective strategy. Existing studies on this topic mainly focus on the strengthening roles of different alloying elements and can generally be classified into two representative alloying strategies. The first involves non-refractory alloying elements [8,9,10,11,12]. Among them, Al is a typical example, whose addition promotes the formation of ordered structures such as the B2 phase, thereby enhancing intermediate-temperature strength through the pinning effect of ordered domains on dislocations. For instance, Yurchenko et al. [13] observed dispersed B2 nano-ordered structures in Al_7.5_(NbTiZr)_92.5_ alloys, which continuously pinned dislocations over the temperature range of 473–873 K and improved the intermediate-temperature strength by approximately 20–30%, demonstrating the pronounced advantage of ordering strengthening in enhancing thermally stable load-bearing capacity. However, excessive ordering may restrict dislocation slip and induce grain boundary embrittlement, resulting in reduced ductility. In contrast, Cu primarily improves mechanical properties through precipitation strengthening. Wang et al. [14] reported that Cu addition in Ti-35Nb-7Zr-10Cu alloys induced the formation of precipitates such as Ti_2_Cu and Cu_8_Zr_3_, which significantly enhanced alloy strength via precipitation strengthening. Nevertheless, these precipitates may also increase microstructural complexity and interfacial mismatch, thereby adversely affecting ductility. The second approach involves refractory alloying elements [15,16]. Cr is a representative example, as its large elastic modulus mismatch provides effective solid-solution strengthening, while its role as a typical β-stabilizing element suppresses the formation of brittle ω phases in Ti-based alloys [17], thereby enhancing strength while helping retain ductility. Zhang et al. [18] tailored the phase constitution and slip behavior of (TiZr)_65−x_Nb_15_Mo_20_Cr_x_ (x = 5, 10, 15, and 20) multi-principal-element alloys through Cr alloying, achieving a compressive yield strength of 712 MPa at 1073 K. Nguyen et al. [19] observed Ta–Nb-rich nanoscale cuboidal regions and Zr-rich nanoscale striped structures in TiZrNbTa medium-entropy alloys; these composition-modulated nanostructures imparted a yield strength of up to 893 MPa at 873 K, indicating that refractory-element-induced nano-heterogeneous structures can effectively retard dislocation recovery and enhance thermal stability at elevated temperatures.

It should be noted that 673 K corresponds to a critical intermediate-temperature service regime for titanium alloys in engineering applications, where materials commonly suffer from enhanced dislocation recovery, altered precipitate stability, and deteriorated strength–ductility synergy. Therefore, 673 K serves not only as a key temperature for evaluating the intermediate-temperature mechanical performance and microstructural stability of Ti-based alloys, but also as an important benchmark for assessing the feasibility of TiZrNb MEAs as engineering substitutes. However, current studies have predominantly focused on either room-temperature properties or higher-temperature regimes (≥873 K), while systematic investigations into the mechanical behavior and deformation mechanisms of TiZrNb-based alloys alloyed with different elements (e.g., Al, Cu, and Cr) at 673 K remain scarce. Accordingly, this work systematically investigates the effects of Al, Cu, and Cr alloying on the mechanical behavior of TiZrNb medium-entropy alloys at room temperature and 673 K to identify the alloy system with optimal performance at 673 K. Combined with strain-rate-dependent mechanical analysis, the intrinsic correlations among dislocation evolution, precipitate stability, and deformation mechanisms are further elucidated.

## 2. Materials and Methods

### 2.1. Material Preparation

TiZrNb, TiZrNb_98.5_Al_1.5_, TiZrNb_98.5_Cu_1.5_, TiZrNb_98.5_Cr_1.5_, TiZrNb_97_Cr_3_ and TiZrNb_95_Cr_5_ alloys were prepared by vacuum arc melting using high-purity Ti (>99.9 wt.%), Nb (>99.9 wt.%), Zr (>99.9 wt.%), Cu (>99.9 wt.%), Al (>99.9 wt.%), and Cr (>99.9 wt.%). The nominal chemical compositions of the alloys are listed in Table 1. To ensure compositional homogeneity, each alloy ingot was flipped and remelted eight times during arc melting. After melting, all six alloy ingots were cold rolled to a total thickness reduction of approximately 70% without any subsequent annealing or heat treatment.

### 2.2. Microstructure Characterization

Phase constitution of the alloys was identified by X-ray diffraction (XRD, Rigaku SmartLab SE, Tokyo, Japan) using Cu Kα radiation. The measurements were conducted over a 2θ range of 10–90° at a scanning rate of 5° min^−1^. The microstructure and elemental distribution of the alloys were characterized using a scanning electron microscope (SEM, ZEISS SUPRA 55, Carl Zeiss AG, Oberkochen, Germany) equipped with an energy-dispersive X-ray spectroscopy (EDS) system. Transmission electron microscopy (TEM, Tecnai F20, FEI, Hillsboro, OR, USA) was employed for detailed characterization of the microstructure and phase structure. TEM specimens were prepared by ion milling using a precision ion polishing system (Gatan 691, Gatan, Pleasanton, CA, USA). The thinning process was carried out at 6 kV and 8° for ~1 h, followed by 5 kV and 6° for ~4 h, and finally at 3 kV and 4° for 10 min to obtain electron-transparent regions suitable for TEM observation.

### 2.3. Mechanical Property Tests

Tensile tests were performed on TiZrNb, TiZrNb_98.5_Al_1.5_, TiZrNb_98.5_Cu_1.5_, and TiZrNb_98.5_Cr_1.5_ alloys at room temperature and 673 K. The tensile specimens were dog-bone-shaped with dimensions of 17.4 mm × 4 mm × 0.7 mm. Room-temperature tensile tests were conducted using a universal testing machine (WDW-200E, Jinan Sida Testing Technology Co., Ltd., Jinan, China), while intermediate-temperature tensile tests were carried out on a high-temperature vacuum testing machine (HT-VAC-1200, Changchun Hengsheng Technology Co., Ltd., Changchun, China). At room temperature, all alloys were tested at strain rate of 1.67 × 10^−3^ s^−1^. At 673 K, tensile tests were conducted at the same strain rate of 1.67 × 10^−3^ s^−1^ under vacuum conditions for TiZrNb, TiZrNb_98.5_Al_1.5_, TiZrNb_98.5_Cu_1.5_, TiZrNb_98.5_Cr_1.5_, TiZrNb_97_Cr_3_, and TiZrNb_95_Cr_5_ alloys. In addition, to investigate the effect of strain rate, TiZrNb and TiZrNb_98.5_Cr_1.5_ alloys were further tested at 673 K with initial strain rates of 1.67 × 10^−3^, 6.67 × 10^−3^, 1.33 × 10^−2^, and 2.67 × 10^−2^ s^−1^. To ensure the reliability of the experimental data, three tensile tests were conducted for each alloy.

## 3. Results and Discussion

### 3.1. Tensile Properties

**Figure 1:**
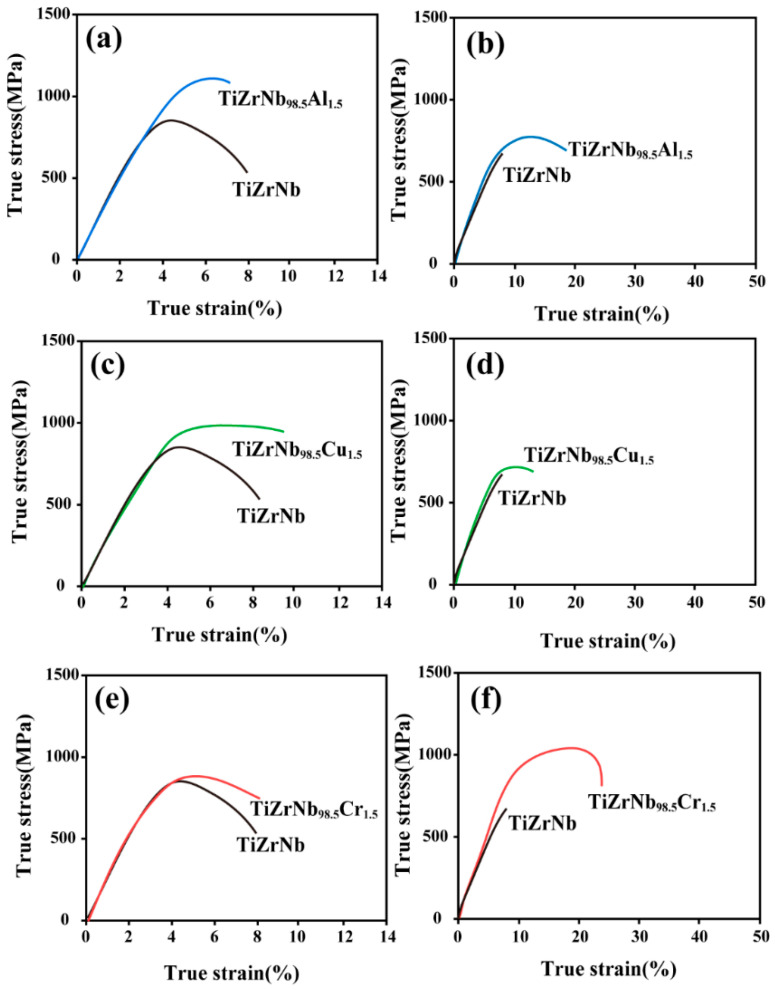
Mechanical properties of TiZrNb alloys alloyed with Al, Cu, and Cr at room temperature and 673 K under a strain rate of 1.67 × 10^−3^ s^−1^. (**a**,**c**,**e**) Room temperature; (**b**,**d**,**f**) 673 K.

As shown in Figure 1a, at room temperature, the TiZrNb_98.5_Al_1.5_ alloy exhibits an ultimate tensile strength of 1125 MPa, which is higher than that of the TiZrNb alloy (906 MPa), but with a concomitant reduction in ductility. This behavior is attributed to the solid-solution strengthening induced by Al addition, which enhances strength at the expense of plasticity [20]. At 673 K, as shown in Figure 1b, the tensile strength of the TiZrNb alloy is 756 MPa, whereas that of the Al-containing alloy increases to 874 MPa with an elongation of 18%. As shown in Figure 1c, Cu alloying simultaneously improves both the strength and ductility of the alloy at room temperature, with the TiZrNb_98.5_Cu_1.5_ alloy exhibiting an ultimate tensile strength of 983 MPa. However, as shown in Figure 1d, the strengthening effect of Cu becomes less pronounced at 673 K. The tensile strength of the TiZrNb_98.5_Cu_1.5_ alloy is 782 MPa, only slightly higher than that of the TiZrNb alloy, indicating a significant reduction in strengthening effectiveness compared with room temperature. As shown in Figure 1e, Cr alloying exerts a relatively limited influence on the mechanical properties at room temperature. The TiZrNb_98.5_Cr_1.5_ alloy exhibits an ultimate tensile strength of 932 MPa, slightly higher than that of the TiZrNb alloy, with an elongation of 8%. In contrast, as shown in Figure 1f, Cr alloying markedly improves the mechanical properties at 673 K. The tensile strength of the TiZrNb_98.5_Cr_1.5_ alloy increases to 1164 MPa, accompanied by an elongation of 25%, demonstrating simultaneous enhancement of both strength and ductility compared with the TiZrNb alloy.

**Figure 2:**
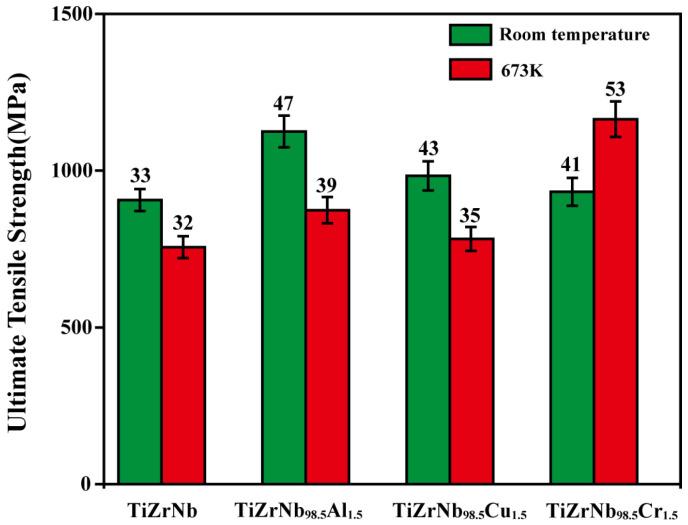
The effects of Al, Cu, and Cr at room temperature and 673 K under a strain rate of 1.67 × 10^−3^ s^−1^ on the strength of TiZrNb.

Figure 2 summarizes the ultimate tensile strengths of TiZrNb, TiZrNb_98.5_Al_1.5_, TiZrNb_98.5_Cu_1.5_, and TiZrNb_98.5_Cr_1.5_ alloys measured at room temperature and 673 K under a strain rate of 1.67 × 10^−3^ s^−1^. At room temperature, the Al and Cu containing alloys exhibit higher strength than the TiZrNb alloy, whereas the Cr-containing alloy shows only a slight improvement in strength. At 673 K, a different trend is observed. The strengthening effects of Al and Cu are weakened, and their strength levels become comparable to that of the TiZrNb alloy. In contrast, the TiZrNb_98.5_Cr_1.5_ alloy exhibits the highest tensile strength (1164 MPa), surpassing all other alloys. These results indicate that, unlike Al and Cu, Cr is effective in simultaneously enhancing the strength and ductility of the TiZrNb alloy at 673 K.

**Figure 3:**
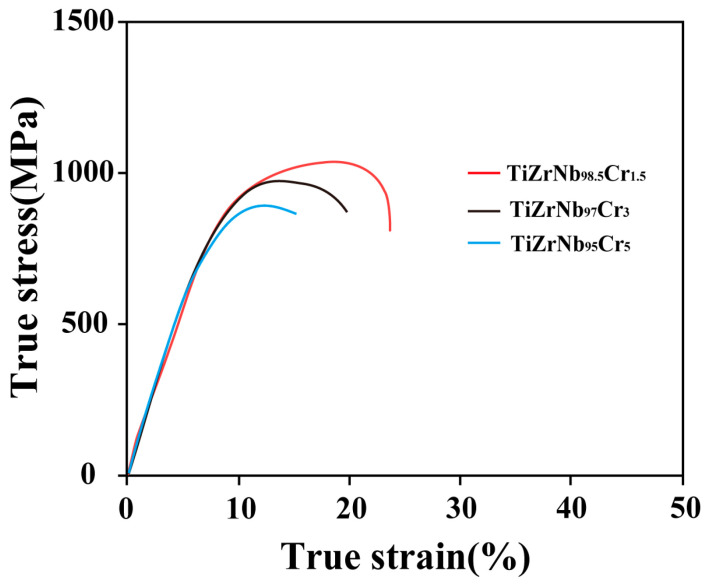
Mechanical properties of the alloys with various Cr contents tested at 673 K under a strain rate of 1.67 × 10^−3^ s^−1^.

To clarify the effect of Cr content on the mechanical properties of the TiZrNb alloy at 673 K, Figure 3 presents the tensile properties of alloys with different Cr additions tested at 673 K and a strain rate of 1.67 × 10^−3^ s^−1^. The results show that the TiZrNb_98.5_Cr_1.5_ alloy exhibits the optimal combination of strength and ductility. With further increasing Cr content, the tensile strengths of the TiZrNb_97_Cr_3_ and TiZrNb_95_Cr_5_ alloys decrease compared with that of the TiZrNb_98.5_Cr_1.5_ alloy. Based on these results, the TiZrNb_98.5_Cr_1.5_ alloy was selected for subsequent systematic investigation in this study.

**Figure 4:**
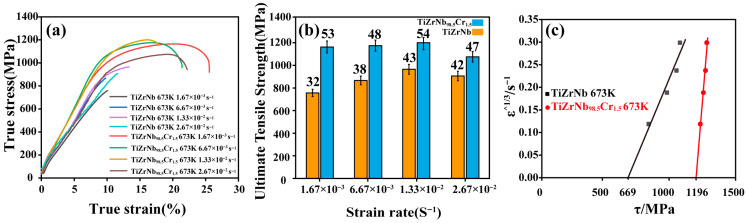
Mechanical Properties of TiZrNb and TiZrNb_98.5_Cr_1.5_ Alloys. (**a**) True stress–strain curves at different tensile strain rates; (**b**) Ultimate tensile strength at different tensile strain rates; (**c**) Threshold stress.

Figure 4 summarizes the tensile properties and threshold stress analysis of TiZrNb and TiZrNb_98.5_Cr_1.5_ alloys at 673 K under different tensile strain rates. As shown in Figure 4a,b, the TiZrNb_98.5_Cr_1.5_ alloy exhibits higher strength and ductility than the TiZrNb alloy at all tested strain rates, indicating that Cr addition improves both the strength and plasticity of the alloy at 673 K. At a strain rate of 1.67 × 10^−3^ s^−1^, Cr addition increases the fracture elongation from approximately 10% to 25%, while simultaneously enhancing the ultimate tensile strength by about 408 MPa. With increasing strain rate, the TiZrNb_98.5_Cr_1.5_ alloy consistently maintains superior overall tensile performance compared with the TiZrNb alloy. Figure 4c presents the threshold stress analysis results for the two alloys. Threshold stress is defined as the minimum effective shear stress required to initiate plastic flow at a given temperature and is typically obtained by linear extrapolation of experimental data [21,22]. The results show that Cr addition increases the threshold stress from 669 MPa for the TiZrNb alloy to 1196 MPa, corresponding to an increase of approximately 79%. The incorporation of Cr enhances the intrinsic resistance of the material to plastic deformation, which is consistent with the higher strength level observed in the TiZrNb_98.5_Cr_1.5_ alloy.

### 3.2. Microstructure

**Figure 5:**
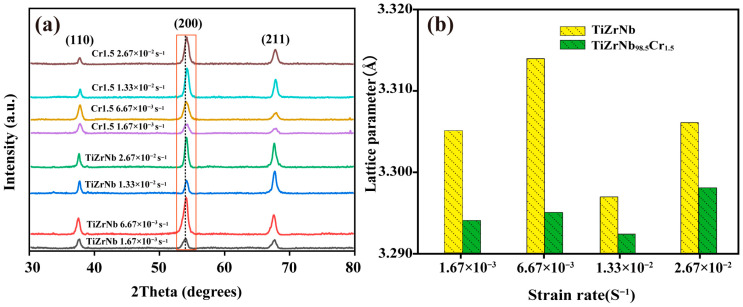
X-ray diffraction patterns of TiZrNb and TiZrNb98.5Cr1.5 alloys deformed at 673 K under different strain rates. (**a**) XRD patterns, the orange box corresponds to the 200 diffraction peak; (**b**) Lattice parameters.

Figure 5 presents the XRD patterns of TiZrNb and TiZrNb_98.5_Cr_1.5_ alloys deformed at different strain rates under 673 K. As shown in Figure 5a, both alloys retain a single body-centered cubic (BCC) phase, consistent with previous reports [7]. Notably, the (200) diffraction peak of the TiZrNb_98.5_Cr_1.5_ alloy shifts toward a higher 2θ angle relative to that of the TiZrNb alloy, accompanied by a reduction in lattice parameter (Figure 5b). This behavior indicates lattice contraction caused by the solid-solution incorporation of Cr into the TiZrNb alloy, where the relatively smaller atomic radius of Cr leads to a reduction in lattice spacing [23].

**Figure 6:**
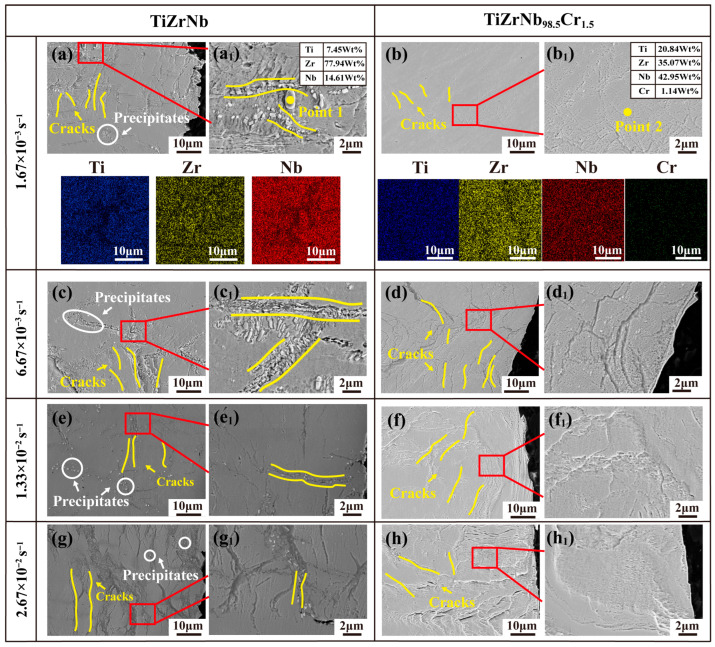
SEM micrographs of TiZrNb and TiZrNb_98.5_Cr_1.5_ alloys deformed at 673 K under various strain rates. (**a**,**c**,**e**,**g**) SEM micrographs of TiZrNb alloy at strain rates from 1.67 × 10^−3^ s^−1^ to 2.67 × 10^−2^ s^−1^; (**a_1_**,**c_1_**,**e_1_**,**g_1_**) Corresponding high-magnification micrographs of TiZrNb alloy at the above strain rates; (**b**,**d**,**f**,**h**) SEM micrographs of TiZrNb_98.5_Cr_1.5_ alloy at strain rates from 1.67 × 10^−3^ s^−1^ to 2.67 × 10^−2^ s^−1^; (**b_1_**,**d_1_**,**f_1_**,**h_1_**) Corresponding high-magnification micrographs of TiZrNb_98.5_Cr_1.5_ alloy at the above strain rates.

Figure 6 shows the SEM morphologies of TiZrNb and TiZrNb_98.5_Cr_1.5_ alloys deformed at 673 K under different strain rates. For the TiZrNb alloy (Figure 6a,c,e,g), distinct particulate precipitates are observed near the crack propagation paths over the strain-rate range of 1.67 × 10^−3^ s^−1^ to 2.67 × 10^−2^ s^−1^. EDS point and mapping analyses reveal that these particles are Zr-rich phases, with a higher Zr concentration in the precipitates than in the matrix. Previous studies have demonstrated that Zr-rich phases located at grain boundaries and within grains are brittle phases that promote crack initiation and accelerate crack propagation during tensile deformation, thereby deteriorating the mechanical performance of the alloy [24,25]. In contrast, for the TiZrNb_98.5_Cr_1.5_ alloy (Figure 6b,d,f,h), no obvious particulate precipitates are observed in the vicinity of cracks throughout the entire strain-rate range. EDS elemental mapping further shows that Ti, Zr, Nb, and Cr are uniformly distributed within the matrix, with no evident elemental segregation. These results indicate that the addition of Cr suppresses Zr segregation and inhibits the formation of Zr-rich precipitates.

**Figure 7:**
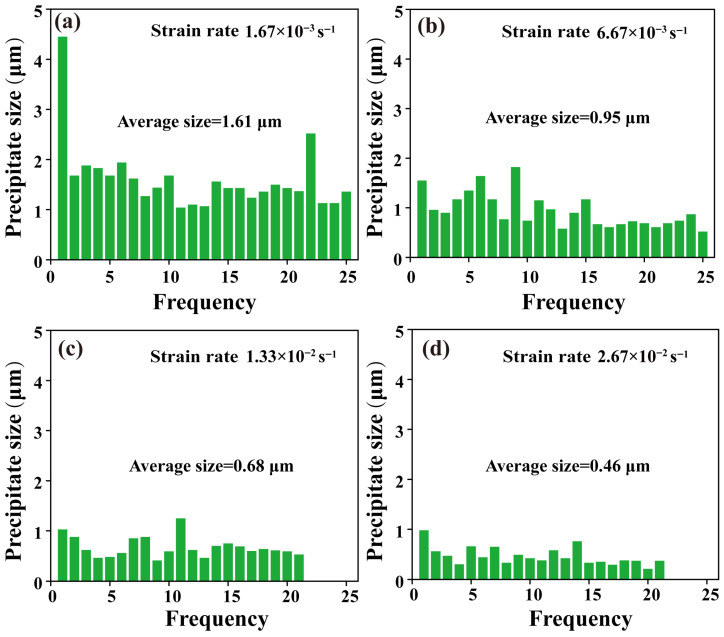
Average size of precipitates in TiZrNb alloy deformed at 673 K under different strain rates. (**a**) Strain rate of 1.67 × 10^−3^ s^−1^; (**b**) Strain rate of 6.67 × 10^−3^ s^−1^; (**c**) Strain rate of 1.33 × 10^−2^ s^−1^; (**d**) Strain rate of 2.67 × 10^−2^ s^−1^.

To further quantitatively characterize the evolution of precipitates in the TiZrNb alloy, statistical analysis of precipitate size under different strain rates was conducted, and the results are presented in Figure 7. At a strain rate of 1.67 × 10^−3^ s^−1^, the precipitates exhibit an average size of 1.61 μm, with the presence of coarse precipitates observed (Figure 7a). When the strain rate increases to 6.67 × 10^−3^ s^−1^, the average precipitate size decreases to 0.95 μm (Figure 7b). With further increases in strain rate to 1.33 × 10^−2^ s^−1^ and 2.67 × 10^−2^ s^−1^, the precipitate size is further refined, and the average size decreases to 0.68 μm and 0.46 μm, respectively (Figure 7c,d). Overall, the average precipitate size in the TiZrNb alloy continuously decreases with increasing strain rate, exhibiting a clear strain-rate dependence.

**Figure 8:**
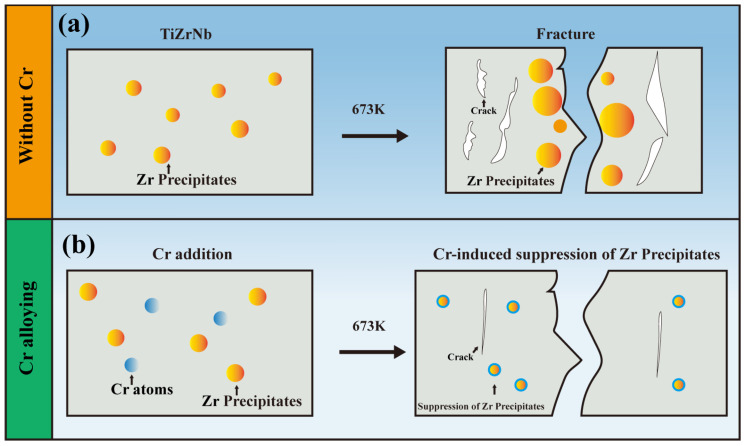
Schematic illustration of Zr precipitation behavior in TiZrNb and TiZrNb_98.5_Cr_1.5_ alloys. (**a**) TiZrNb alloy; (**b**) TiZrNb_98.5_Cr_1.5_ alloy.

To visually compare the differences in Zr-rich precipitate behavior between TiZrNb and TiZrNb_98.5_Cr_1.5_ alloys after tensile deformation at 673 K, schematic illustrations of the two alloys are presented in Figure 8. For the TiZrNb alloy (Figure 8a), Zr-rich precipitates are observed to accumulate along the crack path. In contrast, for the TiZrNb_98.5_Cr_1.5_ alloy (Figure 8b), the introduction of Cr suppresses Zr precipitation, and no aggregation of Zr-rich precipitates is observed after tensile deformation.

To further understand the suppressive effect of Cr on Zr-rich precipitation, an auxiliary analysis of solid-solution stability was conducted using the regular solution model to simplify the free-energy calculation of the multicomponent alloy system. The Gibbs free energy of solid-solution formation in multicomponent alloys is jointly determined by enthalpic and entropic contributions, where the mixing enthalpy (ΔH_mix_) reflects the chemical interactions among constituent elements, and the mixing entropy (ΔS_mix_) characterizes the configurational disorder of the system. A smaller absolute value of ΔH_mix_ favors the formation of a random solid solution, whereas a larger ΔS_mix_ enhances solid-solution stability by lowering the Gibbs free energy of the system [26]. For an n-component alloy system, the mixing enthalpy can be calculated using the following equation [27]:

(1) $$\;\Delta H_{mix}=\sum_{i=1,i\neq j}^{n}4\Delta H_{ij}^{mix}c_{i}c_{j}$$

where $4\Delta H_{ij}^{mix}$ is the interaction parameter between the i-th and j-th elements, $c_{i}$ and $c_{j}$ are the atomic fractions of the i-th and j-th components, respectively, and $\Delta H_{ij}^{mix}$ is the binary mixing enthalpy. In this study, the binary mixing enthalpy values were obtained from the Miedema database [28].

According to Boltzmann’s hypothesis, the mixing entropy of an n-component regular solution model is expressed as follows:

(2) $$\;\Delta S_{mix}=-R\sum_{i=1}^{n}\left(c_{i}lnc_{i}\right)$$

where $c_{i}$ is the molar fraction of each component and R is the gas constant (8.314 J K^−1^ mol^−1^).

Considering that solid-solution formation in multi-principal-element alloys is governed by the combined effects of enthalpy and entropy, a discussion of ΔH_mix_ or alone is insufficient to comprehensively evaluate system stability. Therefore, the Ω parameter was further introduced to characterize the competitive relationship between entropy stabilization and enthalpy driving force, which is defined as follows:

(3) $$\Omega =\frac{T_{m}\Delta S_{mix}}{\left|\Delta H_{mix}\right|}$$

where $T_{m}$ is the average melting point of the alloy, calculated as the atomic-fraction-weighted average of the melting points of the constituent elements:

(4) $${\;T}_{m}=\sum_{i=1}^{n}c_{i}{\left(T_{m}\right)}_{i}$$

It should be noted that the Ω parameter is primarily established based on enthalpy–entropy competition and does not directly account for the influence of atomic size differences on lattice distortion. To further evaluate the contribution of atomic size mismatch to solid-solution stability, the atomic size mismatch parameter δ was introduced, defined as follows:

(5) $$\delta =\sqrt{{\sum_{i=1}^{n}c_{i}(1-\frac{r_{i}}{\bar{r}})}^{2}}$$

where $c_{i}$ is the atomic fraction of the i-th component, and $\bar{r}=\sum_{i=1}^{n}c_{i}r_{i}$ is the average atomic radius. The melting points and atomic radii of Ti, Zr, Nb, and Cr are listed in the Ref. [29].

The calculated results for the two alloys are listed in Table 2. Compared with the TiZrNb alloy, the TiZrNb_98.5_Cr_1.5_ alloy exhibits an increase in ΔS_mix_ from 1.098 R to 1.140 R and an increase in δ from 5.44% to 6.01%, accompanied by a further increase in the Ω parameter, while ΔH_mix_ decreases from 2.57 kJ·mol^−1^ to 1.89 kJ·mol^−1^. These results indicate that the introduction of Cr enhances the configurational entropy of the system and strengthens the lattice distortion effect while reducing the average mixing enthalpy, thereby overall improving the solid-solution stability of the TiZrNb matrix.

**Figure 9:**
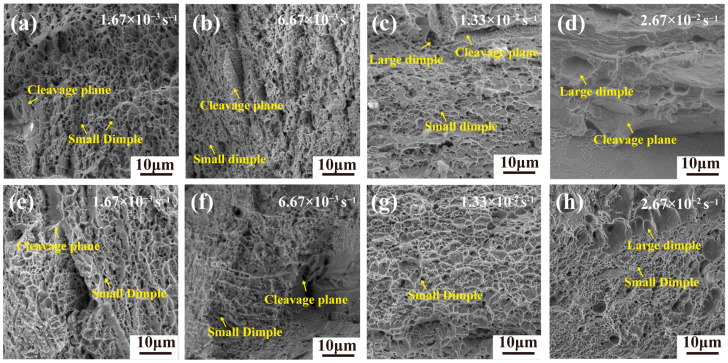
SEM micrographs of the fracture surfaces of the samples deformed at 673 K under different strain rates. (**a**–**d**) TiZrNb alloy at strain rates from 1.67 × 10^−3^ s^−1^ to 2.67 × 10^−2^ s^−1^; (**e**–**h**) TiZrNb_98.5_Cr_1.5_ alloy at strain rates from 1.67 × 10^−3^ s^−1^ to 2.67 × 10^−2^ s^−1^.

Figure 9 shows the SEM micrographs of the tensile fracture surfaces at 673 K under different tensile strain rates. The results (Figure 9) indicate that the TiZrNb alloy exhibits a mixed ductile–brittle fracture mode at all four strain rates. In contrast, the TiZrNb_98.5_Cr_1.5_ alloy shows a mixed ductile–brittle fracture mode at strain rates of 1.67 × 10^−3^ and 6.67 × 10^−3^ s^−1^, whereas with increasing strain rate, it transitions to a predominantly ductile fracture behavior at 1.33 × 10^−2^ and 2.67 × 10^−2^ s^−1^. Distinct fracture morphologies are therefore observed for the two alloys at 673 K. With increasing strain rate, the TiZrNb alloy gradually transforms toward a more brittle fracture mode, while the TiZrNb_98.5_Cr_1.5_ alloy exhibits a reduction in cleavage facets accompanied by an increased density of dimples, indicating enhanced ductile fracture characteristics.

**Figure 10:**
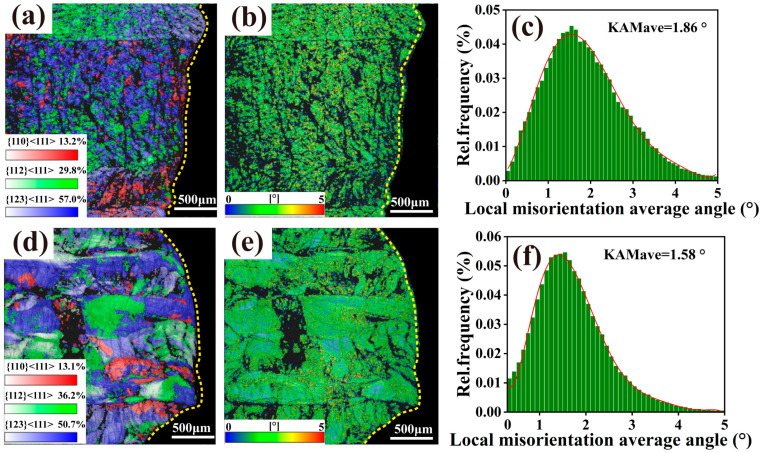
EBSD Schmid factor maps, KAM maps, and statistical plots of average KAM values obtained at 673 K under a strain rate of 1.33 × 10^−2^ s^−1^. (**a**–**c**) TiZrNb alloy; (**d**–**f**) TiZrNb_98.5_Cr_1.5_ alloy.

The EBSD Schmid factor and KAM maps of the TiZrNb and TiZrNb_98.5_Cr_1.5_ alloys after tensile deformation at 673 K and 1.33 × 10^−2^ s^−1^ are shown in Figure 10. As presented in Figure 10a,d, the {110}, {112}, and {123} slip planes were identified in both alloys, among which the {123} slip plane exhibits the highest activation fraction in both cases. This may arise from several factors. In BCC alloys, the occurrence of {123} slip during high-temperature deformation may result from the cooperative activation of multiple {110} and {112} slip systems [30]. In addition, when the critical resolved shear stress (CRSS) for dislocation glide on different slip planes is comparable, the {123} slip system is more likely to adopt mechanically favorable orientations, thereby exhibiting a higher Schmid factor [31]. Elevated temperature combined with low deformation rate also promotes the activation of {123} slip planes [32].

Further analysis of the KAM maps for the two alloys (Figure 10b,e) reveals that the TiZrNb alloy exhibits an overall higher KAM distribution. Its corresponding statistical result (Figure 10c) shows an average KAM value of 1.86°, with high-KAM regions distributed in a relatively dispersed manner. In contrast, the TiZrNb_98.5_Cr_1.5_ alloy develops band-like KAM features aligned along the tensile direction, and its average KAM value decreases to 1.58° (Figure 10f). Since KAM is generally associated with local orientation gradients and lattice distortion, and elevated KAM values are commonly attributed to increased local misorientation induced by dislocation accumulation [33], the higher and more discretely distributed KAM regions in the TiZrNb alloy suggest a tendency toward localized strain concentration during plastic deformation.

**Figure 11:**
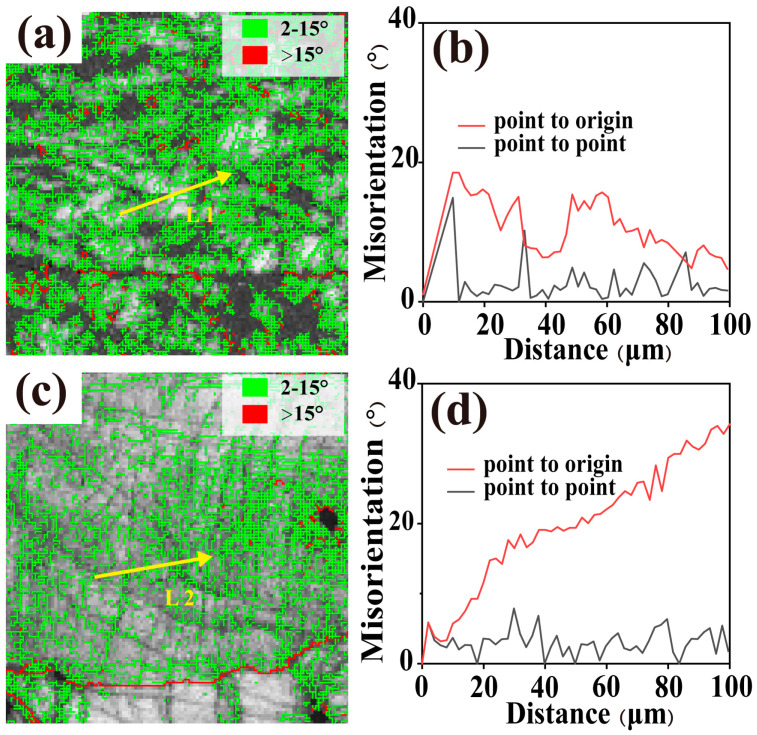
Grain boundary maps and local orientation deviation plots of the alloys deformed at 673 K under a strain rate of 1.33 × 10^−2^ s^−1^. (**a**,**b**) Grain boundary map and local orientation profile along line L1 for TiZrNb alloy; (**c**,**d**) Grain boundary map and local orientation profile along line L2 for TiZrNb_98.5_Cr_1.5_ alloy.

As shown in Figure 11a,b, after tensile deformation at 673 K and 1.33 × 10^−2^ s^−1^, the TiZrNb alloy exhibits a high density of low-angle grain boundaries accompanied by pronounced fluctuations in local orientation, indicating that dislocations do not move uniformly along the entire dislocation line during deformation [34]. In contrast, the TiZrNb_98.5_Cr_1.5_ alloy shows distinctly different characteristics. As presented in Figure 11c,d, the fraction of low-angle grain boundaries is reduced and the fluctuation in local misorientation is less pronounced, suggesting the occurrence of more continuous lattice rotation during deformation. This behavior can be attributed to the introduction of atomic-size-mismatched solute atoms in the BCC alloy, which induces lattice distortion and locally reduces the Peierls stress, thereby enhancing dislocation mobility [35].

**Figure 12:**
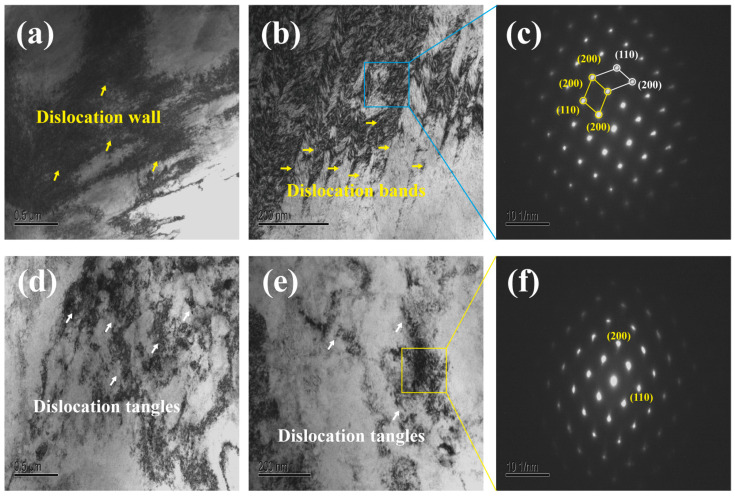
TEM micrographs and SAED patterns at 673 K under a strain rate of 1.33 × 10^−2^ s^−1^. (**a**–**c**) TiZrNb alloy; (**d**–**f**) TiZrNb_98.5_Cr_1.5_ alloy.

Figure 12 presents the TEM results of the TiZrNb and TiZrNb_98.5_Cr_1.5_ alloys after tensile deformation at 673 K and 1.33 × 10^−2^ s^−1^. For the TiZrNb alloy, a high density of dislocations with severe entanglement can be observed, leading to the formation of pronounced dislocation wall structures (Figure 12a). In regions with high dislocation density, dislocation bands are further identified (Figure 12b), and the corresponding SAED pattern exhibits diffraction spots characteristic of a BCC structure (Figure 12c). In contrast, the TiZrNb_98.5_Cr_1.5_ alloy displays a distinctly different deformation substructure. Its overall dislocation density is relatively lower, and dislocations are mainly distributed as discrete entanglements without the formation of obvious dislocation wall structures (Figure 12d,e). Moreover, no dislocation bands similar to those observed in the TiZrNb alloy are detected, while the corresponding SAED pattern likewise confirms the BCC crystal structure (Figure 12f).

The differences in dislocation configurations observed by TEM further reveal distinct substructure evolution behaviors during plastic deformation in the two alloys. The dislocation wall structures formed in the TiZrNb alloy are generally associated with local dislocation accumulation and obstruction by precipitates [36]. Combined with the higher average KAM value (1.86°) and the discretely distributed high-KAM regions identified by EBSD, the TiZrNb alloy exhibits pronounced local strain heterogeneity during deformation. In comparison, dislocations in the TiZrNb_98.5_Cr_1.5_ alloy are predominantly distributed as discrete entanglements, consistent with its lower average KAM value (1.58°) and more homogeneous KAM distribution.

## 4. Conclusions

This study systematically investigates the effects of Al, Cu, and Cr alloying on the tensile properties of TiZrNb alloy at 673 K, with particular emphasis on the regulatory role of Cr in microstructural evolution and deformation behavior at this temperature. The main conclusions are summarized as follows:

(1) Cr alloying exhibits a unique strengthening effect in TiZrNb medium-entropy alloys at 673 K compared with Al and Cu additions. While Al and Cu improve room-temperature strength, their strengthening effects diminish markedly at elevated temperature. In contrast, TiZrNb_98.5_Cr_1.5_ achieves the best mechanical performance at 673 K, with an ultimate tensile strength of 1164 MPa and elongation of 25%.
(2) Cr addition effectively suppresses Zr segregation and inhibits the formation of brittle Zr-rich precipitates during deformation at 673 K. In the TiZrNb alloy, abundant Zr-rich precipitates accumulate along crack propagation paths, promoting crack initiation and propagation. By contrast, no obvious precipitate aggregation is observed in TiZrNb_98.5_Cr_1.5_, and EDS mapping confirms a more homogeneous elemental distribution, indicating enhanced microstructural stability during tensile deformation.
(3) Cr addition significantly modifies the deformation microstructure of the TiZrNb alloy at 673 K by promoting more homogeneous plastic deformation and suppressing localized strain concentration. Compared with TiZrNb alloy, the TiZrNb_98.5_Cr_1.5_ alloy exhibits reduced deformation heterogeneity and a more uniform intragranular strain distribution, while its dislocation substructure is characterized by discretely distributed dislocation tangles without pronounced dislocation wall formation. In contrast, the TiZrNb alloy shows severe dislocation accumulation accompanied by dislocation wall structures and localized dislocation bands.

## Figures and Tables

**Table 1:** Chemical composition of the bulk samples of TiZrNb_98.5_(Al,Cu,Cr)_1.5_ Alloys in at.%.

Samples	Ti	Zr	Nb	Al	Cu	Cr
TiZrNb	32.1	33.2	34.7			
TiZrNb_98.5_Al_1.5_	31.62	32.7	34.18	1.5		
TiZrNb_98.5_Cu_1.5_	31.62	32.7	34.18		1.5	
TiZrNb_98.5_Cr_1.5_	31.62	32.7	34.18			1.5
TiZrNb_97_Cr_3_	31.14	32.2	33.66			3
TiZrNb_95_Cr_5_	30.5	31.54	32.97			5

**Table 2:** Values of mixing enthalpy (ΔH_mix_), mixing entropy (ΔS_mix_), Ω parameter and atomic size mismatch parameter (δ) for TiZrNb and TiZrNb_98.5_Cr_1.5_ alloys.

Samples	ΔH_mix_ (kJ mol^−1^)	ΔS_mix_ (R)	Ω	δ (%)
TiZrNb	2.57	1.098	8.11	5.44
TiZrNb_98.5_Cr_1.5_	1.89	1.140	11.44	6.01

## Data Availability

The original contributions presented in this study are included in the article. Further inquiries can be directed to the corresponding author.
